# Genetic Analysis of Low BMI Phenotype in the Utah Population Database

**DOI:** 10.1371/journal.pone.0080287

**Published:** 2013-12-11

**Authors:** William R. Yates, Craig Johnson, Patrick McKee, Lisa A. Cannon-Albright

**Affiliations:** 1 Laureate Institute for Brain Research, Tulsa, Oklahoma, United States of America; 2 University of Oklahoma College of Medicine, Tulsa, Tulsa, Oklahoma, United States of America; 3 Eating Recovery Center, Denver, Colorado, United States of America; 4 University of Oklahoma College of Medicine, Oklahoma City, Oklahoma, United States of America; 5 Department of Medicine (Genetic Epidemiology), University of Utah School of Medicine, Salt Lake City, Utah, United States of America; 6 George E. Wahlen Department of Veterans Affairs Medical Center, Salt Lake City, Utah, United States of America; Sanjay Gandhi Medical Institute, India

## Abstract

The low body mass index (BMI) phenotype of less than 18.5 has been linked to medical and psychological morbidity as well as increased mortality risk. Although genetic factors have been shown to influence BMI across the entire BMI, the contribution of genetic factors to the low BMI phenotype is unclear. We hypothesized genetic factors would contribute to risk of a low BMI phenotype. To test this hypothesis, we conducted a genealogy data analysis using height and weight measurements from driver's license data from the Utah Population Data Base. The Genealogical Index of Familiality (GIF) test and relative risk in relatives were used to examine evidence for excess relatedness among individuals with the low BMI phenotype. The overall GIF test for excess relatedness in the low BMI phenotype showed a significant excess over expected (GIF 4.47 for all cases versus 4.10 for controls, overall empirical p-value<0.001). The significant excess relatedness was still observed when close relationships were ignored, supporting a specific genetic contribution rather than only a family environmental effect. This study supports a specific genetic contribution in the risk for the low BMI phenotype. Better understanding of the genetic contribution to low BMI holds promise for weight regulation and potentially for novel strategies in the treatment of leanness and obesity.

## Introduction

Genetic factors increase the risk for a high body mass index (BMI), overweight and obesity [1]. The role of genetic factors in low BMI is less well understood.

Family studies have found family clustering for low BMI [2]–[4]. However, family studies cannot distinguish between genetic and familial factors. Twin studies have estimated the heritability of BMI across the entire BMI range at between 50 and 74% [5]–[6].

Molecular genetic studies have identified a series of candidate genes for low BMI including a thyrotropin-releasing hormone (TRH) receptor polymorphism [7], the Ser23 allele of the serotonin 2C receptor [8], a genetic variant on chromosome 16p11.2 [9] and a copy number variant identified as gremlin1 [10]. Allelic variants of the *FTO* gene linked to obesity risk are infrequently found in thin individuals [11].

Understanding the genetic and environmental contributions to low BMI are important because low BMI has been linked to medical and psychiatric illnesses as well as increased mortality. In females, low BMI during childhood and adolescence increases women's risk for later endometriosis [12], preterm birth [13], low infant birth weight [14] and increased risk for placental abruption [15]. Infants born to mothers with low BMI have an increased risk for atrial septal defect, genital abnormalities including hypospadias [16].

The psychiatric illness *anorexia nervosa* is defined by a low BMI in association with extreme fear of becoming fat [17].

Mortality rates across BMI categories in many studies display a U-shaped curve with increased death rates for the low BMI as well as those with a high BMI. Mortality rates are higher by an estimated 73% in those with BMI below 18.5 [18]. A Japanese study estimated the mortality risk increased by 78% in those with a BMI<18.5 and increased by 155% in those with a BMI<16 [19]. The exact mechanism for the mortality increase in low BMI populations is unclear. Some of the increase may be due to higher rates of death in severe illness and surgical procedures such as lung transplantation [20].

A study of excess deaths related to being low BMI and high BMI in the United States provides a reference for the relative contribution of low BMI to mortality [21]. High BMI was estimated to contribute to 111,909 deaths in the U.S. in 2000 while low BMI was estimated to be associated with 33,746 deaths. Thus, low BMI is estimated to contribute about three deaths for every ten deaths related to being high BMI.

To further understand the prevalence and genetic contributions of leanness, we examined the low BMI phenotype in the Utah Population Data Base (UPDB). The UPDB provides a strategy to examine the possibility of both environmental and genetic contributions to a phenotype by estimating risk in both close and distant relatives and by testing for excess familial clustering. An observation of excess close relationships alone would not have allowed discrimination between shared genes and shared environment, but the UPDB allows us to consider more distant relationships that are unlikely to represent lifestyle sharing beyond what is expected in the Utah population. We hypothesized that low BMI individuals would demonstrate near and distant familial clustering consistent with a genetic contribution.

## Methods

### Ethics Approval

The protocol for this study was approved by the University of Utah Institutional Review Board and the Resource for Genetic and Epidemiologic Research. The Resource for Genetic and Epidemiologic Research is the oversight board for the Utah Population Data Base. All data used in this research study contains no individual identifiers. A waiver of consent was approved for this study due to the lack of individual identifiers for all subjects. Consent requirements were waived for this study since obtaining consent would have unnecessarily identified individuals in the anonymous database. Review of this protocol by the University of Utah Institutional Review Board and the Resource for Genetic and Epidemiologic Research includes a review and approval of consent issues and other ethical aspects of the research.

### Utah Population Data Base (UPDB)

The UPDB is a unique computerized database primarily representing the pioneer founders of Utah and their modern day descendants. It includes up to 15 of genealogy data dating back to the original Utah founding pioneers [22], as well as current generations. The genealogy data has been linked to statewide data including driving license (DL) data, births, deaths, the Utah Cancer Registry, and Utah Hospital Discharge Data, among other data sets (www.huntsmancancer.org/groups/ppr). The DL data includes height and weight and is available for over three million Utah drivers.

For the genetic analyses performed here we selected only from those 1,192,768 individuals in the UPDB who have genealogy data for both parents, all four grandparents, and six of their eight great grandparents and whose genealogy connects to the original Utah genealogy, and the 593,704 of these individuals who have Utah Drivers License data. These strict criteria allow for appropriate matching of cases and controls in terms of quality and quantity of genealogical data.

The oversight board for the UPDB encourages collaboration with outside investigators and institutions. Researchers with interest in using the UPDB to test hypotheses may contact the board or one of the authors for information about methods to apply for access.

The UPDB has been successfully used to define familial clustering and genetic influences in a variety of disorders including cancer [23]–[27], coronary artery disease [28], diabetes [29] rotator cuff disease [30], and deaths due to influenza [31] and asthma [32]. The methods used to identify phenotypes, assess familial and genetic effects and identify pedigrees using UPDB data have been described in detail in these studies. The study of high-risk pedigrees identified in the UPDB has led to multiple gene identifications, including *BRCA1* [33], *BRCA2* [34], *CDKN2A* (melanoma) [35]–[36] and *HPC2/ELAC2* [37].

### Low BMI phenotype

The phenotype of adult leanness was established using Utah DL data available for 593,704 individuals (with acceptable genealogy data as described) included in the UPDB. We identified all male and female drivers whose most recent calculated BMI (from height and weight provided) was <18.5. Rates for the low BMI phenotype were calculated by age group and are shown in Table 1, which includes the age group, the number of lean individuals in the age group, the total number of individuals with DL data in the age group, the leanness prevalence and the 95% confidence interval for prevalence by age group, estimated by the method of Clopper and Pearson [38].

**Table 1:** Prevalence Rates for Low BMI (<18.5) in the UPDB.

Age	BMI<18.5	N	Prevalence (%)	95% CI
15–19	6,259	57,010	11.0	10.7, 11.2
20–24	2,847	62,070	4.6	4.4, 4.8
25–29	1,629	63,548	2.5	2.4, 2.7
30–34	922	49.069	1.9	1.8, 2.0
35–39	544	39,353	1.4	1.3, 1.5
40–44	400	36,097	1.1	1.0, 1.2
45–49	311	40,719	0.8	0.7, 0.9
50–54	258	42,998	0.6	0.5, 0.7
55–59	176	38,040	0.4	0.3, 0.5
60–64	135	26,947	0.5	0.4, 0.6
65–69	191	27,076	0.7	0.6, 0.8
70–74	255	26,207	1.0	0.9, 1.1
75–79	354	25,122	1.4	1.3, 1.6
80 or older	569	26,979	2.1	1.9, 2.3

### Statistical Analysis

The Genealogical Index of Familiality (GIF) statistic was used to test the hypothesis of excess relatedness among individuals in the low BMI phenotype. The GIF was developed specifically for the UPDB [39]–[40]. Briefly, the GIF measures the average pair-wise relatedness of a set of individuals and compares that measurement to the average pair-wise relatedness expected in the Utah population. The GIF test differs from relative risk (RR) in that it includes analysis of all genetic relationships, both close and distant. The GIF utilizes the Malecot coefficient of kinship to measure pair-wise relatedness. The coefficient is defined as the probability that randomly selected homologous genes from two individuals are identical by descent from a common ancestor [41]. The coefficient is 0.50 for parent/offspring, 0.25 for a sibling pair, 0.125 for an uncle/nephew pair, 0.0625 for a first cousin pair, and so forth. The contribution to the GIF statistic is therefore smaller for individual pairs with greater genetic distance between them; more closely related pairs contribute more.

To evaluate the significance of the GIF test, we estimated the average pair-wise relatedness for 1,000 sets of controls matched to the cases by birth year, sex, and birthplace (Utah or not). These controls are chosen from among the 593,704 individuals with acceptable quality genealogy data who also have DL data. The empirical significance of the GIF test is measured by comparing the case GIF to the distribution of 1,000 control GIF values.

The GIF statistic measures familial clustering, which can be due to genetic (genes related to low BMI phenotype), or to shared familial environmental effects (i.e. familial preference for low calorie diet or rigorous physical exercise), or to a combination of both. In order to better distinguish these effects, we recalculate the case GIF and the control GIFs while ignoring close relationships (first and second degree). If this distant GIF (dGif) test is significant, it provides strong evidence that there is significant distant excess relatedness that is unlikely to be due to shared environment.

The calculation of RR in relatives provides the more traditional mechanism for identifying genetic effects. A genetic contribution to a phenotype is supported when both close and distant relatives have elevated risk. RRs for the low BMI phenotype were estimated for first-, second- and third degree relatives of low BMI individuals as follows. First-degree relatives include parents, siblings and offspring; second-degree relatives are the first-degree relatives of the first-degree relatives (e.g. uncle, grandmother); third-degree relatives are the first-degree relatives of the second-degree relatives (e.g. first cousin, great grandchild), All 593,704 individuals in the UPDB with acceptable quality and quantity genealogy data as described and with DL data were assigned to one of 132 cohorts based on birth year (in five year groups), sex, and birthplace (Utah or not). Cohort-specific rates of low BMI were estimated by dividing the total number of low BMI individuals per cohort by the total number of individuals with DL data per cohort. Expected numbers of low BMI first-degree relatives were estimated by counting the number of first-degree relatives with DL data and genealogy data by cohort (without duplication), multiplying by the rate of low BMI in each cohort, and summing over all cohorts. Observed numbers of low BMI individuals (BMI<18.5) among relatives were counted without duplication. RRs were estimated for each degree of relationship as observed/expected number of low BMI individuals; 95% confidence intervals for the RR were calculated using the method of Agresti [42]

### High-risk Pedigree Identification

It is possible to identify pedigrees in the UPDB with a significant excess of low BMI using the same tools listed above. We first identify all possible related clusters of individuals with low BMI; no cluster is a subset of any other cluster, but individuals can be identified in more than 1 cluster. These clusters represent all sets of related individuals with low BMI descending from a common founder (a pedigree), but they are not necessarily high-risk for low BMI, they can represent chance clusters. To identify which of the clusters are high-risk for low BMI, we apply the internal cohort-specific rates for low BMI we estimated from the UPDB (see above methods for Relative Risks) to all of the descendants of each cluster. We compare the observed number of low BMI cases among the descendants to the expected number of low BMI cases among the descendants to identify those pedigrees with a significant excess of low BMI cases.

## Results

### Prevalence Rates and Proband Selection

The prevalence of BMI<18.5 in the UPDB individuals with acceptable quality and quantity genealogy are summarized by age group in Table 1. The prevalence of BMI<18.5 ranged from .4% in the 55 to 59 year old age group to 11.0% in those aged 15 to 19 years. The BMI in the sample ranged from a minimum of 12.10 to a maximum of 62.93.

Table 1 illustrates some developmental factors associated with weight: i) stable adult weight often is not reached until midlife; ii) younger individuals have different ranges in BMI as they progress through adolescence and young adulthood, and iii) in older geriatric age groups, rates of low BMI increase with the loss of lean body mass with aging. To minimize phenotypic heterogeneity due to these other factors, we elected to include individuals between the ages of 25 and 64. This age period showed relatively stable low BMI population prevalence rates and occurred outside the periods of early and late adult development. We identified 4,375 individuals between the ages of 25 and 64 years with BMI<18.5 with acceptable quality genealogy data.

### Genealogy Index of Famililiality (GIF)

The GIF test for excess relatedness in low BMI was performed on all of the 14,867 low BMI individuals and the results were compared to the average relatedness observed among 1,000 sets of matched controls selected from all individuals who had a Utah driver's license. Table 2 shows the number of cases, the average relatedness of the cases, the mean control relatedness, the empirical p value for the test for excess overall relatedness, and the empirical p value for the test for excess distant relatedness. The overall GIF test for excess relatedness for everyone with low BMI shows a significant excess over expected (p-value<0.001). The distant GIF test, ignoring close relatives (genetic distance<4) also showed a significant excess (p-value = 0.031).

**Table 2:** GIF Test for Low BMI (<18.5) in the UPDB.

Group	N	Case GIF	Control GIF	GIF p-value	dGIF p-value
All BMI<18.5	14,867	4.47	4.10	<0.001	0.031
BMI<18.5, ages 25–64	4,375	4.84	4.19	<.0.001	<0.001

**Figure 1:**
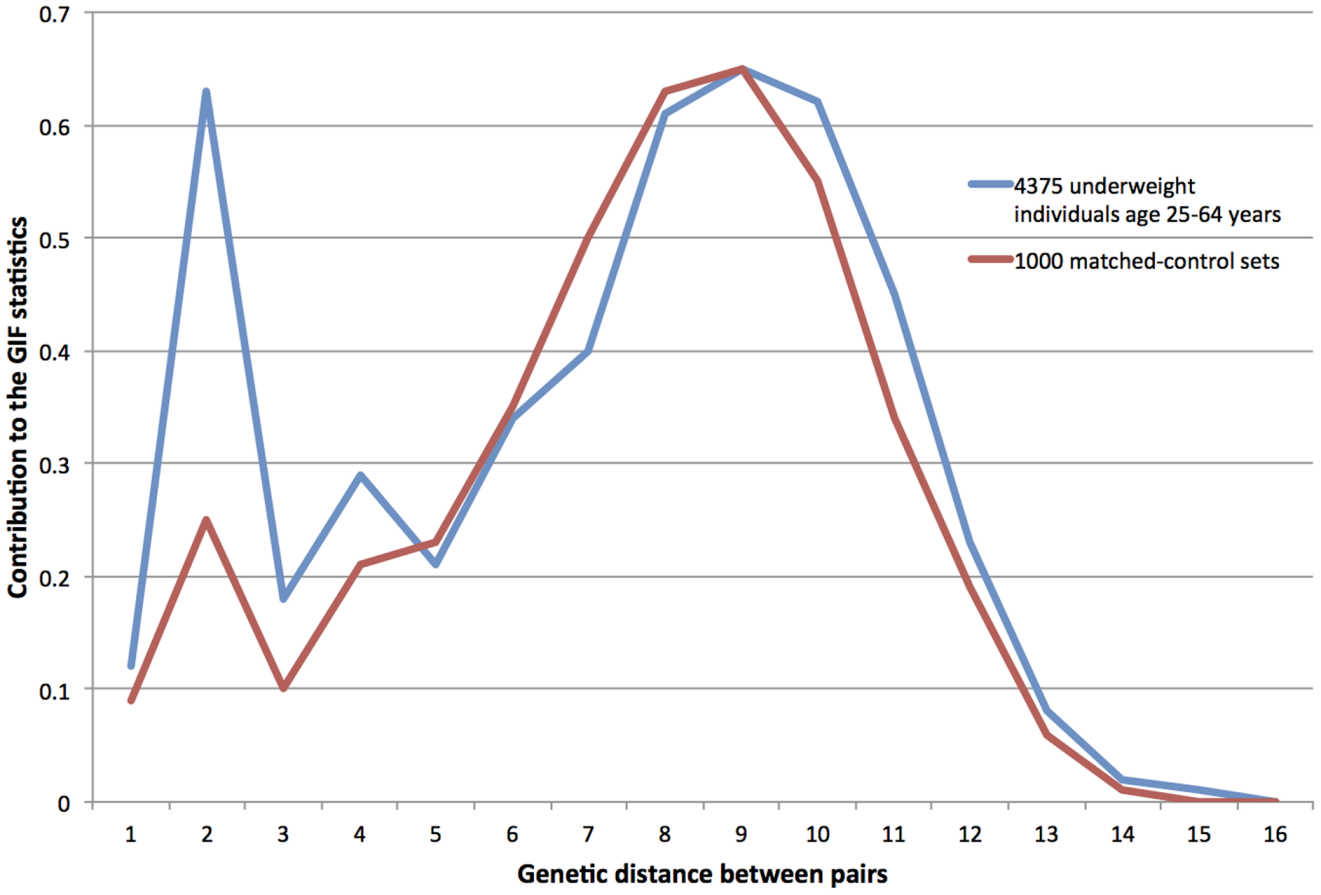
The contribution to the GIF statistic by genetic distance for 4,375 low BMI cases aged 25–64 years old compared to 1,000 sets of matched UPDB controls with BMI data. Genetic distance between pairs is shown on the x-axis and represents an increasing measure of relatedness (1 = parent/offspring; 2 = siblings, e.g.; 3 = uncle/niece, e.g.; 4 = first cousins, e.g.) from close to distant; the most distant relationships noted (genetic distance = 16) could represent, for example, two individuals who have a common ancestor 8 generations past. The cumulative contribution to the GIF statistic for each relatedness (as measured by genetic distance) for all pairs identified at that genetic distance is represented on the y-axis. The contribution to the GIF statistic for each larger genetic distance is one-half as large; the contribution for genetic distance 1 = ½, for genetic distance 2 = ¼, and so forth. The distribution for controls represents the expected relatedness of a group of individuals just like the cases (ignoring BMI) and is smoother because it is averaged over 1,000 different sets of controls tested. The distribution for cases represents only the analysis of the single set of cases and is more irregular. The peak at genetic distance = 2 (e.g. siblings) in comparison with genetic distance = 1 (parent/offspring) is seen for both cases and controls and represents that we observe more sib pairs than parent/offspring pairs in our data. A similar peak for cases at genetic distance 4 also indicates that we observed more cousins (same generation) than avunculars, for example.

Table 2 also shows the results of the GIF test for the 4,375 low BMI adults aged 25–64 years of age. Similar results were obtained for this subgroup of lean individuals from which individuals at the extremes of the age distribution were removed to reduce bias. Figure 1 shows the contribution to the GIF statistic, separately for cases and controls, by pairwise genetic distance where genetic distance 1 = parent/offspring, 2 = siblings, 3 = avunculars, 4 = first cousins, and so forth for the low BMI individuals aged 25–64 years. As seen, the distribution of relatedness for cases is in excess up to genetic distance = 4 (e.g. first cousins) and beyond genetic distance = 9 (e.g. second-cousins once-removed. Although the pairwise relatedness distributions for cases and matched controls cross at some points, as seen in Figure 1, the pairwise relatedness for cases is significantly elevated over that for matched controls when all genetic distances are considered as determined by distant Gif test (dGif p = 0.031).

### Relative Risks (RRs)

Estimates of relative risks in relatives of lean adults are shown in Table 3. The table shows the total number of each type of relative with BMI data (# relatives), the observed number of those relatives with BMI<18.5 (obs), and the expected number of relatives with BMI<18.5 (expected) based on birth year, sex, and birthplace cohort-specific rates for low BMI among all UPDB individuals with BMI data.RRs for adult leanness were significantly elevated among first-, second-, and third-degree relatives of lean adults. The smaller number of second degree lean adults observed is not unexpected, given that second degree relatives are primarily in different generations (avunculars, grandparent/child), while first and third-degree relatives occur in the same generation (siblings and cousins, respectively) as well as in different generations (parent/offspring). Since the DL data exist only after 1980, there is a very narrow window that limits observations across generations; however, our results support the GIF results, where the contribution from first- (genetic distance 1 and 2), second- (genetic distance = 3) and third-degree relatives (genetic distance = 4) was in excess for cases compared to controls. Table 4 shows the RR for leanness among the relatives of those lean Utah adults who were aged 25–65 years. Although the RR estimates are slightly larger, the conclusions are not different.

**Table 3:** Relative Risk of Low BMI (<18.5) in Relatives of All Lean Utah Adults.

Relatives	N	Observed	Expected	p-value	Relative Risk	95% CI
First-degree	54,324	3,565	1,615.5	<0.00001	2.21	2.13, 2.28
Second-degree	96,151	2,236	1,799.7	2.1 e-23	1.24	1.19, 1.30
Third-degree	175,286	5,204	4,546.0	7.7 e-22	1.14	1.11, 1.18
Fourth-degree	317,807	6,897	6,773.4	0.133	1.02	.99, 1.04
Fifth-degree	501,420	12,417	12,271.5	0.191	1.01	.99, 1.03
Sixth-degree	571,643	14,180	14,147.9	0.788	1.00	.99, 1.02
Seventh-degree	588,223	14,796	14,772.6	0.849	1.00	.99, 1.02

**Table 4:** Relative Risk of Low BMI (<18.5) in Relatives of Lean Utah Adults Ages 25–65 Years.

Relatives	N	Observed	Expected	p-value	Relative Risk	95% CI
First-degree	20,551	1,177	458.0	<0.00001	2.57	2.42, 2.72
Second-degree	32,849	1,056	775.4	<0.00001	1.36	1.28, 1.45
Third-degree	63,856	1,668	1,363.8	7.0 e-16	1.22	1.17, 1.28
Fourth-degree	153,915	3,597	3,427.9	0.0039	1.05	1.02, 1.08
Fifth-degree	309,372	6,899	6,774.1	.130	1.02	0.99, 1.04
Sixth-degree	474,053	11,512	11,376.2	.204	1.01	0.99, 1.03
Seventh-degree	562,037	12,984	13,955.3	.809	1.00	0.99, 1.02

### Pedigree Identification

We identified all possible clusters of the 4,375 low BMI individuals between 25 and 65 years of age. These clusters merely represent all related sets of individuals with low BMI, they are not necessarily high-risk for BMI. We further evaluate each cluster by testing for an excess of low BMI among all the descendants of the founding pair of the cluster using the low BMI rates estimated from the UPDB. We identified over 4,000 clusters of low BMI relatives, ranging in size from 2 related cases (n = 789 clusters) to size 168 (n = 1 cluster).

**Figure 2:**
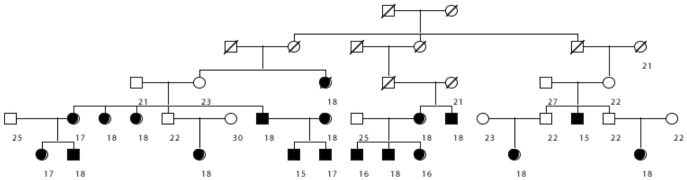
Example UPDB pedigree with a statitstical excess of low bmi (<18.5) individuals. Individuals with bmi <18.5 are fully shaded and BMI is shown beneath subjects where available.

We were able to identify thousands of individual pedigrees that may assist in future molecular genetic studies of the low BMI phenotype. As an example, we have identified 63 pedigrees with a significant excess of individuals with low BMI (p<0.0001) with at least 10 cases. An example of one of these pedigrees is shown in Figure 2. As can be observed, Utah driver's license data is only available for the most recent two or three generations of the Utah genealogy; earlier generations remain unknown for the phenotype of interest.

## Discussion

Familial clustering of the low BMI phenotype in the UPDB is consistent with both genetic as well as environmental contributions to low BMI humans. Increased relative risks for the low BMI phenotype in first-, second-, and third-degree relatives suggests that genetic factors contribute to the familial clustering pattern.

The GIF analysis confirms a genetic contribution to low BMI in relatives with an even more distant degree of relatedness. Excess relatedness among close relatives could represent either shared environment or shared genetics, or a combination,. However, the finding of excess relatedness in distant relatives is much more likely to result from shared genes than shared environment.

Our study represents the largest genealogical population genetics study of low BMI to date. There are no comparable low BMI studies using a similar methodology. However, our identification of a genetic contribution to low BMI is consistent with findings in family studies [2]–[4] and in a single adoption study [43].

A primary limitation of this study is the reliability and validity of Department of Motor Vehicle height and weight self-reported measures. There is limited study of the accuracy of self-reported height and weight in DL data. Self-reported weights in overweight and obese individuals might be significantly underestimated due to the social stigma of obesity. In contrast, social stigma issues in reporting an accurate weight in the low BMI may be less than in obesity. Nevertheless, it remains possible that there is some social pressure to overestimate weight among the low BMI.

Utah driver's license BMI data from the Utah Population Data Base has been compared to BMI data obtained by the CDC Behavioral Risk Factor Surveillance System (BRFSS). This analysis found BMI means generally varied in males by only three percent between the databases with no bias toward over or underestimation across age categories. In younger female age groups (between 25 and 34 years), BMI means from Utah driver's license data was 5 to 8% lower than means from the BRFSS [44].

We compared rates of low BMI in the UPDB to the National Health and Nutrition Examination Survey (NHANES) to look for evidence of a self-report bias. [45]–[46]. The NHANES includes data from direct measurement of weight and height in a representative sample of individuals in the United States. The overall NHANES estimated prevalence of low BMI (BMI<18.5) is 1.8% in the U.S. adult population.

Prevalence rate estimates of low BMI in the UPDB generally are in close agreement with estimates from the NHANES. Rate estimates for BMI<18.5 by age group for the UPDB and (NHANES) were:20 to 39 years of age, 3.3%, (2.6%), 40 to 59 years of age, 0.9%, (1.2%), 60 to 74 years of age, .9%, (.9%), 75 years of age and older, 2.0%, (1.7%). This agreement supports the validity of the low BMI phenotype in the UPDB. Nevertheless, there is no easy method to directly measure the reliability and validity of the drivers license self-reported weight and height in the UPDB.

Some degree of inaccurate self-report of weight may be present and contribute to variance in our relative risk estimates. However, weight self-report bias would likely lead to underestimation of the relative risk.

Identification of familial relationships in the UPDB is also based on self-report. It is possible, self-reported relationships may differ from biological relationships. However, thousands of UPDB high-risk pedigrees have been genotyped with a very high rate of accuracy between self-reported relationships and genotype.

The UPDB cohorts cross several generations. Environmental dietary and physical exercise patterns likely change across generations and may have influenced a portion of BMI data.

Despite these potential limitations, this study finds excess clustering of the low BMI phenotype among both close and distant relatives supporting a significant genetic contribution. Environmental factors are also likely to contribute to familial clustering of low BMI. Families often share diet, exercise and other lifestyle patterns that contribute to body weight.

Pedigrees in the UPDB with a significant excess of low BMI individuals among the descendants of founder couples have been identified. These pedigrees could prove valuable for additional molecular genetic studies of low BMI. Figure 2 shows an example pedigree with a significant excess of individuals with low BMI. The pedigree founder has 257 descendants with BMI data. Eighteen of these individuals have a BMI<18.5. This is significantly greater than 5.3, the number expected in the pedigree (p = 1.13 e^5^).

Further studies to confirm our results and to explore the molecular genetics of the low BMI phenotype are needed. Such studies may uncover the mechanisms for increased morbidity and mortality in low BMI populations. Additionally, further understanding of the genetic and environmental contributions to low BMI may provide insight for prevention and treatment of both low BMI as well as obesity.
